# Bridging the gap with a gender lens: How two implementation research datasets were repurposed to inform health policy reform in Kenya

**DOI:** 10.1093/heapol/czaa117

**Published:** 2020-11-06

**Authors:** Lauren Suchman, Gabrielle Appleford, Edward Owino, Charlotte Avery Seefeld

**Affiliations:** 1 Evaluation Director, Institute for Global Health Sciences, University of California, San Francisco; 2 Consultant Director, Ridge Lane Associates, Nairobi, Kenya; 3 Independent Consultant, Nairobi, Kenya; 4 Program Coordinator, Institute for Global Health Sciences, University of California, San Francisco

**Keywords:** gender, health insurance, policy analysis, research methods, research to policy, Kenya

## Abstract

Policies as they are written often mask the power relations behind their creation (Hull, 2008). As a result, not only are policies that appear neat on the page frequently messy in their implementation on the ground, but the messiness of implementation, and implementation science, often brings these hidden power relations to light. In this paper, we examine the process by which different data sources were generated within a programme meant to increase access to quality private healthcare for the poorest populations in Kenya, how these sources were brought and analyzed together to examine gender bias in the large-scale rollout of Kenya’s National Hospital Insurance Fund (NHIF) beyond public hospitals and civil service employees, and how these findings ultimately were developed in real time to feed into the NHIF reform process. We point to the ways in which data generated for implementation science purposes and without a specific focus on gender were analyzed with a policy implementation analysis lens to look at gender issues at the policy level, and pay particular attention to the role that the ongoing close partnership between the evaluators and implementers played in allowing the teams to develop and turn findings around on short timelines. In conclusion, we discuss possibilities for programme evaluators and implementers to generate new data and feed routine monitoring data into policy reform processes to create a health policy environment that serves patients more effectively and equitably. Implementation science is generally focused on programmatic improvement; the experiences in Kenya make clear that it can, and should, also be considered for policy improvement.

**KEY MESSAGES**

1. Data generated through implementation science can be used to speak to broader issues related to policy.
2. Taking a community-based participatory research (CBPR) approach to developing and conducting implementation science studies is suggested for future research to generate findings that meaningfully bring the perspective of healthcare providers on the ground to the policy level.

## Introduction

### Background

Universal Health Coverage (UHC) cannot be achieved without attention to equity. However, equity often is equated to socioeconomic status with less attention to other social stratifiers. Research has found that unless policy makers pay explicit attention to gender, efforts to reach UHC may not improve equity and may in fact exacerbate existing gender inequities (Witter *et al.*, 2017). When it comes to health care, women incur more out-of-pocket expenditure than men, which is due in part to women’s specific health needs related to pregnancy, childbirth, contraception and abortion among others (WHO, 2010). Over the past few decades, low- and middle-income countries (LMICs) have started to institute social and community-based health insurance schemes to make healthcare more accessible to the general population by lowering out-of-pocket costs (Spaan *et al.*, 2012). However, many of these schemes cover only those working in the formal sector and their dependents, or require the payment of monthly premiums. Since most women in sub-Saharan Africa are employed in the informal sector, this precludes them from being a primary beneficiary in many national health insurance schemes. Further, the unpredictable wages common to this sector can hamper their ability to pay the monthly premiums required to maintain coverage (Chuma *et al.*, 2012; ILO, 2018).

While middle-income countries such as Thailand and Taiwan have long since achieved UHC using national health insurance (Tangcharoensathien *et al.*, 2018; Hsiao *et al.*, 2019), many LMICs attempting to reach UHC in line with Sustainable Development Goal 3.8. are either implementing these schemes for the first time or revamping pre-existing schemes to better serve their wider population. As these programmes roll out, attention to gender equity is crucial to ensure that no is one is left behind in the UHC agenda. Policy implementation analysis will be important for countries to undertake in order to determine where roadblocks exist for women and how to course correct along the way.

### Gender and health systems

Health systems are not gender neutral, often reinforcing restrictive social norms that place women at a disadvantage compared to male counterparts (Hay *et al.*, 2019). Indeed, women and men have different health care needs. However, while many health systems attempt to address the different biological differences between women and men through, for example, essential benefits packages, there is a tendency to assume that maternal health programmes are an adequate response to address most major gender differences in health (WHO, 2010). A common solution to ensuring healthcare is accessible to all is providing free services (Kabia *et al.*, 2018). However, this solution does not address all of the issues that many women, especially poor and disabled women, have accessing care, nor does eliminating fees, by itself, make the healthcare system equal. Larger social issues, such as transportation costs, access to employment, and women’s role within the family all affect women’s access to care (Morgan *et al.*, 2016). A number of studies have found that, beyond biological differences, gender affects healthcare experiences from time spent seeking care (Yeatman *et al.*, 2018) to the ability to access care when caring for others (Kabia *et al.*, 2018).

Beyond women’s experiences as patients, we also know that women make up a significant proportion of both the formal and informal health workforce (Hoss *et al.*, 2011; Harman, 2016) and these numbers are increasing in LMICs, paralleling shifts in high-income countries (Russo *et al.*, 2015). However, in clinical practice and in academia, the leadership is highly skewed toward men (Exavery *et al.*, 2013; Dhatt *et al.*, 2017). Indeed, as some studies have shown, gender parity in the healthcare workforce may lead to better health outcomes for patients, but larger structural changes are necessary to foster a health system that is gender equitable (Hay *et al.*, 2019).

Evidence on women and health insurance tends to be split between: (1) the determinants of who is most likely to be insured or uninsured, with gender being one possible factor; and (2) service utilization by those who are insured vs. those who are uninsured. This evidence generally shows that women, the poor, and uneducated individuals are less likely to be insured than men, individuals from high socioeconomic groups and those with formal education (Kimani *et al.*, 2014; Brugiavini and Pace, 2016). Further, individuals who have health insurance are more likely to seek health services than individuals who do not (Dixon et al., 2014) and some studies have found a positive correlation between insurance enrollment and health outcomes (Mostert *et al.*, 2014). Much of the literature on women in this area focuses on maternal health, suggesting that women who have access to health insurance are more likely to use formal maternity services (Smith and Sulzbach, 2008) and may have better health outcomes as well (Hawks *et al.*, 2018). However, this narrow focus on maternal services may mask larger challenges: work examining general gender disparities under India’s Rashtriya Swasthya Bima Yojana (RSBY) scheme has demonstrated that women have a harder time accessing national health insurance due to a number of factors. These include gender inequities that restrict their mobility and the limit of five RSBY registrants per household, which can lead to larger families prioritizing men and boys for insurance access over women and girls (Cerceau, 2012). However, we did not find any similar literature on gender disparities and women’s experiences using national health insurance in sub-Saharan Africa, suggesting that this is an area of inquiry that should be explored further as a number of countries implement such schemes in the pursuit of UHC.

### Implementation science vs. Policy implementation analysis

Implementation science generally is understood to bridge the so-called “knowledge-to-practice gap” that exists between the development of evidence-based practices, the communication of these practices to professionals in the field, and the extent to which these professionals put their knowledge to practical use (Eccles and Mittman, 2006). Indeed, research has shown that failure to adequately bridge the knowledge-practice gap affects health outcomes across both high-income countries and their low- and middle-income counterparts (Glasziou *et al.*, 2017), making “knowledge translation” critical to improving healthcare quality (LaRocca *et al.*, 2012). However, implementation science often has a relatively narrow focus and the policy environment within which a particular health programme or facility functions often is considered a contextual factor that may influence implementation, rather than the subject of study itself (Damschroder *et al.*, 2009; Lau *et al.*, 2016).

Policy implementation analysis is similar to implementation science in some of its methodological approaches, as well as its desire to improve health outcomes by identifying areas where translating established protocols into practice falls apart. However, policy implementation analysis differs in its object of study (policy vs. clinic-based intervention), its (non)use of theory, its limited ability to establish causal pathways between protocol and practice, and its embrace of complexity (Nilsen *et al.*, 2013). Further, it tends to focus on high-income countries with little policy implementation analysis having been conducted in low- and middle-income settings (Saetren, 2005).

This paper aims to apply a policy implementation lens to data collected for implementation sciences purposes in an LMIC (Kenya). It offers one example of a programme in which data routinely collected for implementation science purposes at the clinic level was supplemented with data on the larger policy environment to draw conclusions about gender equity in policy implementation. The programme studied was a complex intervention package that drew on national health insurance accreditation to both improve quality at the clinic level and increase income for small private providers. In addition to routine clinic-level data collection by an external evaluation team as well as the programme implementers themselves, both the evaluators and implementers collected data on the larger policy environment within which the programme functioned. Noticing gender disparities in their respective datasets, the evaluation and implementation teams worked together to generate findings around gender bias in the policy implementation process. These findings resulted in a joint policy brief presented to Kenya’s Health Financing Reforms Panel on the Transformation and Repositioning of the National Hospital Insurance Fund as a Strategic Purchaser towards the attainment of Universal Health Coverage.

### Programme and policy context

The data for this paper were collected by two different teams, an external qualitative evaluation team and an implementation team, working with the African Health Markets for Equity (AHME) programme in Kenya. AHME was initiated in 2012 and concluded in March 2019. The programme aimed to link healthcare supply (small and medium enterprise (SME) private providers) with demand (clients) in order to shift health markets toward providing quality healthcare to populations living in poverty in Kenya and Ghana. The AHME partners included: Marie Stopes International (MSI); Marie Stopes Kenya (MSK); Population Services Kenya (PSK); Population Services International; Marie Stopes Ghana; and the PharmAccess Foundation. Past partners included: the International Finance Corporation; Society for Family Health, Nigeria; and the Grameen Foundation. AHME worked through social franchises, networks of providers that apply the principles of commercial franchising to health services (Schlein and Montagu, 2012), to provide a package of quality improvement and financing interventions. This package included: social franchising; SafeCare, a step-wise quality improvement programme managed by the PharmAccess Foundation; the Medical Credit Fund, a business training and loans programme also managed by the PharmAccess Foundation; and National Health Insurance accreditation assistance. On the demand side, AHME also provided support for activities to identify and enroll low-income populations into National Health Insurance. Funding for AHME was provided by the Bill and Melinda Gates Foundation and the UK Department for International Development.

The National Hospital Insurance Fund (NHIF) is one of the key vehicles for UHC in Kenya and played a critical role in both supply- and demand-side financing for AHME. The NHIF was established in 1966 with a core mandate to provide health insurance cover to all its members and their dependents. During the AHME implementation period, the Kenyan government funded special programmes under the NHIF to increase access to insurance for people living in poverty, the elderly, secondary school children, and pregnant women, in addition to rolling out SupaCover, a programme specifically meant for informal sector workers. However, coverage in the NHIF remains limited due to the predominance of the informal sector in Kenya, which was estimated to include 83% of Kenya’s population in 2017 (NHIF, 2017). While NHIF coverage is mandatory for all formal sector workers (Government of Kenya, 2012), it remains voluntary for the informal sector despite the fact that the 1998 Amendment to the NHIF Act requires that all Kenyans have health insurance. At the end of 2018, <20% of the population was estimated to be covered by the NHIF (Barasa *et al.*, 2018). Even when nominally covered, true enrollment is often low: out of 2.9 million members from the informal sector, only 988 662 members were active or current in payment as at 30th June 2017, which represents a retention rate of 27% (Barasa, 2019).

In response to the many shifts in NHIF policy during the AHME implementation period, the programme was required to adapt and adjust its policy interventions. Originally, the policy objectives of AHME’s demand-side financing work were to support the Kenyan government to test and scale the Health Insurance Subsidy Programme (targeted to poor populations) and increase voluntary enrolment for those in the two lowest wealth quintiles into NHIF. These policy objectives evolved over time in response to both the government’s new initiatives within NHIF, and the changes within the AHME programme. On the supply side, given its programmatic focus on SME providers, AHME worked to increase the number of accredited SME private providers, which was believed to align with increased quality of care, in addition to building providers’ business skills and capacity to help them better manage NHIF contracts.

## Methods

This paper draws from two separate datasets collected under the African Health Markets for Equity (AHME) programme. One of these datasets was collected by the AHME Qualitative Evaluation team from the University of California San Francisco as part of the AHME programme evaluation in Kenya. The other was collected by the AHME partners for the purposes of internal monitoring, evaluation and learning.

### The AHME qualitative evaluation

As part of the AHME Qualitative Evaluation in Kenya, semi-structured interviews were conducted with private healthcare providers and women exiting private clinics; these were both AHME-supported and non-AHME clinics. Using a purposeful criterion sampling strategy (Palinkas *et al.*, 2015) to ensure a range of experiences with the AHME interventions, sample clinics were selected from lists of franchised facilities provided by the AHME partners. The Qualitative Evaluation team partnered with Innovations for Poverty Action (IPA), a research organization based in New Haven, CT USA with country offices across the globe to collect data in four rounds: 2013, 2015, 2017 and 2018. In order to align with data collected for the Quantitative Evaluation, women exiting the sample clinics were screened for eligibility according to: sex (women only); age (between 18-49 years of age); and number of children (interviewees were required to have at least one child aged 5 years or less). Clients also were selected for NHI enrollment status with an aim to sample NHI-enrolled and non-enrolled patients equally. Across rounds of data collection, providers were asked about their experiences with the AHME intervention package, their experiences working with the AHME partners themselves and, in later rounds, opportunities and challenges around NHIF accreditation. Clients were asked about the quality and accessibility of services in the clinics. In later rounds of interviews (2017 and 2018), clients with NHIF coverage were specifically asked about their experience using NHIF in the clinic, while patients without coverage were asked to describe what they knew about the scheme. In total, 173 providers and 86 patients were interviewed.

Interview recordings were translated from Swahili and transcribed simultaneously by a team of professional transcriptionists. The UCSF team then coded the transcripts with some assistance from IPA using Atlas.ti, a widely-used qualitative analysis software package. Since there was little existing literature on private provider and patient experiences with the NHIF from which to derive prior theories, the team used an inductive thematic approach to coding and analysis. Codes were refined over the several rounds of analysis to allow for new priorities while ensuring continuity, and research team members reviewed the codebook together in each round to ensure consistency in code application.

The UCSF team received initial approval with “Exempt” status from the Institutional Review Board of the University of California San Francisco for the AHME evaluation on 13 June 2013. In addition, the team received Ghana Health Services Ethical Review Committee (ERC) approval on 28 June 2013 and Kenya Medical Research Institute (KEMRI) approval on 28 October 2013. Prior to each round of data collection the Qualitative Evaluation team submitted amendments and received approval from all three review boards for any changes made to our protocol. Approvals for Round 3 (2018) of data collection was received on 15 June 2018 from ERC, 22 May 2018 for UCSF, and 10 May 2018 for KEMRI.

### Internal AHME data collection

AHME learnings were curated by the implementing partners through a series of case studies and other learning products, which were based upon analysis of AHME secondary data as well as primary data collection. Primary data collection entailed site visits and key informant interviews with private providers and NHIF branch managers. Data were collected over three years in 2016, 2017 and 2018/19. The focus of key informant interviews was to generate learning around the experiences and perceptions of private providers in NHIF schemes; the nature and value of the support provided by AHME to private providers; and areas for improvement for the effective participation of private providers in NHIF schemes.

### Bringing the data together

The AHME programme enjoyed a well-funded mixed-methods external evaluation that was underpinned by a learning agenda shared across both the evaluation and implementation teams. As such, programme evaluators and implementers maintained regular communication regarding data collection and the production of learning products. However, the learning agenda was not designed to include findings specifically related to gender disparities and very few learning products were envisioned to include co-authors from more than one team or to triangulate data across teams.

As findings around the relationship between gender and access to meaningful NHIF coverage began to emerge from data routinely collected at the clinic level for the qualitative evaluation team, members of the two teams began to discuss these findings and their relevance to data collection the implementers were undertaking to learn more about the policy environment and its effects on AHME-supported providers. Members from both teams met several times to discuss their respective findings. While the triangulated data were not sufficient to produce a joint academic paper, an opportunity arose for the two teams to use the data that were already available to write a joint policy brief for the NHIF reforms committee that began meeting in June 2019. Working on a short timeline, the evaluators and implementers were able to combine their respective datasets and draft a short policy brief, including recommendations for addressing gender inequities embedded in the NHIF system.

## Results

To generate joint findings, the qualitative evaluation team from UCSF contributed analysis of in-depth interviews (IDIs) conducted with both private providers and their patients over four rounds of data collection. Internal AHME programme data also included qualitative data (IDIs), which were triangulated with AHME monitoring data and complemented by desk review of relevant documents (such as NHIF guidelines, contracts, etc.). Both teams collected data for implementation science purposes to support programme learning and adaptation, although the evaluation team was tasked with providing an assessment of barriers and facilitators to the success of the AHME intervention package, while the implementers had an additional interest in supporting the NHIF and providers to engage more effectively and efficiently. Further, the qualitative evaluation had more breadth insofar as it included a greater number of respondents. Internal programme learning had more depth in that it explored specific, emergent, operational and procedural issues from a provider perspective and used this lens to interact with government and other stakeholders.

After combining their respective datasets, the evaluation and implementation teams produced a joint policy brief for the NHIF reforms committee that included the following preliminary findings and resulting recommendations.

### Understanding of family planning coverage under the NHIF is limited in practice

Although family planning is critical to women’s sexual and reproductive health, unmet need for family planning services in Kenya remains high (Kaneda and Greenbaum, 2019). As learned from AHME, the inclusion of family planning services within the NHIF benefits package is not well understood by providers or by NHIF members, and is often characterized by inequities in service entitlements; this creates an additional barrier to access for women seeking modern contraceptive methods. For example, permanent methods, namely tubal ligation and vasectomy, are included as part of the benefits package and are paid on a fixed fee-for-service^1^ for those under the enhanced medical schemes, while this benefit is excluded under other schemes that are more widely accessible to the broad population. All other family planning methods, including long-acting reversible contraception (LARC), such as implants and intrauterine contraceptive devices, are included in the out-patient national scheme, paid through capitation.^2^ While capitation was selected as the preferred means of provider payment ‘to induce positive incentives in the health delivery system’ (Government of Kenya, 2012), this may not be the case for family planning services. Upon discovering that clients were having an especially difficult time accessing LARCs using their NHIF coverage, the AHME implementers undertook a case study with private providers that focused on the effects of NHIF tariffs on private provider businesses. This analysis ultimately determined that, in many instances, private providers in the NHIF are unclear about the inclusion of family planning services under capitation (Appleford and Owino, 2017).

Similarly, the AHME qualitative evaluation team asked private providers about their experiences serving patients after becoming NHIF-accredited. From these interviews, it became clear that private providers typically understand capitation to be a “cap” on the cost of services they are allowed to offer, rather than a facility-level risk pool in which those patients who use cheaper services or none at all balance out the patients who require more costly services. In response, providers either limit services or charge patients unnecessarily for more expensive services and supplies.

*Interviewer: So, what do you do if somebody came and maybe spend like seven hundred [Kenyan Shillings], and you are allowed not to take more than three hundred per quarter [under capitation]? What do you do?*

*Respondent: Sometimes if the balance is too big we force them to pay.*

(Finance Officer at a private clinic, Nyanza, Kenya)

Thus, even when providers understand how facility-level risk pooling works, capitation encourages providers to offer cheaper, easier to administer methods. Since LARC methods require more time, skills and consumables, providers are therefore discouraged from offering a comprehensive contraceptive method mix under this financing strategy. While capitation is meant to contain costs, learning from AHME therefore suggests that contraception may be ill-suited to capitation financing.

Further, as learned through an internal AHME case study conducted in early 2019 through site visits to selected private providers, there is limited understanding of the inclusion of post-partum family planning services in the Linda Mama (free maternity services) package by both private providers and women patients (Appleford, 2019). While post-partum family planning services are included as part of postnatal care (PNC), the reimbursement rate for each PNC visit is flat and does not reflect the cost of offering this service. Linda Mama is recognised in Kenya’s FP2020 ‘Actions for Acceleration’ as an immediate opportunity to improve access to post-partum family planning. However, without a differential reimbursement for this service, it is unlikely that providers will pro-actively offer the service to patients.

### NHIF registration and use of coverage are more challenging when a husband is not present

Through AHME, the Amua and Tunza social franchises supported awareness creation and public education activities around the Linda Mama and Supacover schemes aimed specifically at informal sector workers. This entailed NHIF branch offices, social franchise providers and community mobilisers conducting joint SupaCover membership drives and other outreach events. Through these drives, the AHME implementers learned that in some regions, men were considered by default the head of household and therefore the principal NHIF member. This was found to stall registration into the NHIF if the male head of household was not present when registration events took place.

Similarly, when conducting IDIs with clients exiting private health facilities, both the AHME qualitative evaluation team and the implementing partners asked women about their NHIF membership status and their use of NHIF cards to pay for services. Findings from these interviews suggest that a number of women who have NHIF coverage do not always use this coverage when they visit health providers, which is partly due to the NHIF’s policy of issuing only one card to a household’s principal contributor to cover an entire family. Since the principal contributor is most often male, many women do not have direct access to their NHIF membership card or may not know their membership number to confirm coverage when visiting a health provider. As the AHME implementers found through literature review and interviews with key informants, although NHIF enrollees are identified through a biometric system at the point of care, biometric registration is not required upon enrollment into the NHIF and the NHIF registration system is not integrated with other government-run biometric systems. This makes it difficult to identify beneficiaries at the point of care unless they are carrying an NHIF membership card. So, while biometric registration should theoretically allow enrollees to use their NHIF coverage any time without carrying a membership card, in practice women often have to be accompanied by their husband as the principal cardholder in order to access health services.

Some evidence from the qualitative evaluation suggests that women may also face challenges using their NHIF coverage as a result of economic or seasonal migration that separates families. If women are living separately from their husbands, this limits their access to the household’s NHIF membership card, as well as to the health facility to which the entire family is capitated.

*Interviewer: And which hospital did you choose?*

*Respondent: Here I have not chosen, eeh because that time I was in Mombasa. So that card itself is with…with my husband, eeh.*

*Interviewer: So, you didn’t use it?*

*Respondent: I have never used it again apart from that time when I was admitted [in Mombasa].*

(Client at a private clinic, Embu County)

In addition to women not being able to physically access their NHIF membership card when living separately from their husband, capitation and the associated lack of portability of benefits may play a role in limiting access, as primary cardholders are most likely to capitate to a health facility that is geographically convenient for themselves.

In order for healthcare coverage under NHIF to be truly universal, gendered inequities in service access, the disproportionate barrier that out-of-pocket expenditure creates for women and the needs of women and adolescent girls need to be understood and *effectively* addressed. As a result of the above findings, the AHME evaluation and implementation teams recommended a change in the mode of reimbursement of LARC methods under the Supacover scheme to motivate health facilities to provide these services, thereby ensuring women have access to family planning, as well as a range of method choices. In addition, the teams recommended that NHIF cards be issued to all members, not just the principal contributor in each household.

## Discussion

The nature of implementation science is such that data gathered under this umbrella can have broad applicability beyond the programme level and can be used to speak to issues that are relevant to policy development and reform. While the AHME programme contained a policy component from the beginning, the data presented here from the qualitative evaluation team was collected for the purposes of monitoring the implementation of a clinic-based intervention. In addition to their own internal monitoring at the clinic level, the programme implementers also collected data on the larger policy environment in Kenya to feed into planned policy work as well as to better understand the ways in which AHME-supported providers were affected by changes at the policy level. Together, the evaluators and implementers were therefore able to use data meant for programme implementation monitoring to measure the indirect effects of policy implementation and the effects of regulatory shifts on SME private providers and their patients. The resulting findings, which point to structural challenges that inhibit women’s access to and use of key reproductive health services like family planning under a national health insurance scheme, are critical points to consider if the Kenyan government wishes to meaningfully pursue UHC.

To achieve this end, the evaluation and implementation teams maintained regular communication and a shared learning agenda, which helped to facilitate data sharing and eventual triangulation. However, the learning agenda was created relatively late in the course of the programme and, as such, it did less to guide the separate teams in planning jointly produced learning products and acted more as an internal tracking system. The NHIF gender policy brief, for example, was never included in the shared learning agenda. In addition, the realization of the learning agenda was complicated by delays in the AHME quantitative evaluation, which made it virtually impossible for the other teams to triangulate data with this piece of the external evaluation.

Regarding internal communications, we note that implementers often are reluctant to share too much with external evaluators out of fear of receiving a negative evaluation that could have consequences for funding and programme sustainability. In the case of AHME, both the qualitative evaluation and implementation teams benefited from the programme’s relatively long timeline, which allowed them to establish trust and develop more open communication. It was this open communication that made the analysis presented in this paper possible, rather than a formal plan to share and triangulate data. As a result, we suggest that developing a joint learning agenda early in a project’s implementation and evaluation phase would be beneficial for other programme’s hoping to generate shared findings. This learning agenda must then be underpinned by mutual trust and shared timelines that allow for the free and timely flow of information among teams.

In addition to developing strong internal programme communication and coordination to re-tool implementation science data for policy implementation analysis, we recommend that programme recipients be engaged to conduct more robust analyses. In the case described above, the AHME evaluators and implementers were in a privileged position to feed the results of their analysis directly into a government process due to the relationships they had forged and the status they enjoyed at the highest levels of government. While implementation data collection could be most efficient and resulting analysis most relevant at the provider level if both were led by providers themselves, or at least undertaken with greater involvement from providers, as we have shown elsewhere (Sieverding *et al.*, 2018; Suchman *et al.*, 2018) SME private providers tend to have little or no voice in the larger health system and we know little about their experiences under programmes like social health insurance. Unlike in high-income countries, where insurance schemes often necessitate that private providers become part of a larger provider network, this is not the case in Kenya. Although they make up a significant proportion of the health market in LMICs (Shah *et al.*, 2011), SME private providers tend to operate independently with little connection to the larger health system outside of social franchise networks (Shroff *et al.*, 2018). As a result, when healthcare change processes are under way, such as the NHIF reforms, it is challenging for these providers to participate meaningfully in such processes. In Kenya, for example, current discussions around the UHC agenda tend to include members of provider associations that primarily draw their membership from larger private and faith-based hospitals, leaving SME private providers virtually unrepresented in UHC discussions. Without an obvious gateway through which SME providers can voice their concerns at the policy level, these concerns will continue to be sidelined in health system reform processes.

While some SME private providers are taking matters into their own hands and establishing their own professional associations, such as Kenya’s newly formed Rural and Urban Private Health Association (RUPHA), moving forward it will be important to develop more effective mechanisms through which these providers and the research that involves them can feed back into the larger health system. To this end, researchers and programme implementers working with these provider populations can do their part to increase the visibility and influence of SME private providers. As in the case of AHME, researchers and programme implementers gathering data for implementation science purposes may be able to apply a policy implementation analysis lens to their data, drawing out lessons in real time as programmes progress. Capturing and sharing these lessons gives weight to the experiences of SME private providers and helps to create a unified voice for a disparate group. In addition, researchers can take a Community-Based Participatory Research (CBPR) approach (Minkler and Wallerstein, 2008) and tap into professional associations such as RUPHA to jointly develop research protocols and priorities. Some scholars suggest that using a CBPR approach is in fact particularly desirable for implementation research (Di Ruggiero and Edwards, 2018) and certainly using CBPR can create more equitable relationships between researchers and participants that in turn generate better, more relevant data (Palinkas, 2019). As in the case of AHME, researchers can then leverage the privileged position in which they sit to amplify SME providers’ perspectives at the policy level. When the voices of these providers are heard at the level of government, this can help to create policies that are more inclusive of all providers’ needs, in turn allowing them to better serve their patient populations more effectively and equitably, as required on the journey to UHC.

## Conclusion

Due to their sustained collaboration, regular communication and shared learning agenda, the AHME qualitative evaluation and programme teams were able to recognize shared patterns of gender inequity in data meant for programme implementation monitoring. As a result, the two teams were able to apply a policy implementation analysis lens to their respective datasets and bring them together into a policy brief, recommending policy changes that could affect women’s access to healthcare in the NHIF reforms process. The teams were able to leverage pre-existing relationships developed with the NHIF to introduce this brief to the official NHIF reforms committee. The insights into gender detailed above are an example of how additional perspectives can see and communicate new ideas back to programme and policy makers, short-circuiting communication systems that may inhibit rapid feedback in order to understand and improve policies as they roll out. Since SME private providers like those represented in AHME make up a significant proportion of the provider landscape in many LMICs, but rarely have a voice in the larger health system, programme evaluators and implementers collecting routine monitoring data should consider applying policy implementation analysis to their data where possible. In addition, researchers working with SME private providers should use newly-formed provider associations as a resource to conduct Community-Based Participatory Research. These are efficient ways to generate findings that are relevant and applicable to policy makers, and prompt reforms that enable these providers to offer more effective and equitable care in the pursuit of UHC.

Finally, it is important to note that the AHME programme was not designed with either a gender or policy implementation analysis lens. Both emerged through interaction of the evaluation and implementation teams as it became increasingly evident that barriers to NHIF participation were engendered for women participating in the scheme. A policy window emerged through the NHIF reforms committee to address these issues, which the two teams have worked to exploit. This work remains ongoing. While emergent, the authors feel that the findings and discussion are timely. More deliberate analysis of gender disparities should inform future evaluation and programmatic work on UHC.

## Footnotes

1 Fixed payment by service, case, or day, triggered when services are delivered (Holtz and Sarker, 2018)

2 Capitation is a payment arrangement for health care service providers that pays a set amount for each enrolled person assigned to them, per period of time, whether or not that person seeks care (Holtz and Sarker, 2018)
